# Protective Effect of Fermented Camel Milk Containing *Bifidobacterium longum* BB536 on Blood Lipid Profile in Hypercholesterolemic Rats

**DOI:** 10.1155/2021/1557945

**Published:** 2021-10-29

**Authors:** Khaled M. El-Zahar, Mohamed F. Y. Hassan, Suliman F. Al-Qaba

**Affiliations:** ^1^Department of Food Science & Human Nutrition, College of Agriculture and Veterinary Medicine, Qassim University, Buraydah 51452, Qassim, Saudi Arabia; ^2^Food Science Department, Faculty of Agriculture, Zagazig University, Zagazig 44511, Egypt; ^3^Department of Dairy Science, Faculty of Agriculture, Sohag University, Sohag, Egypt

## Abstract

The present study aimed to investigate synergistic health effects of camel milk and *Bif. longum* BB536 in rats with diet-induced obesity, impaired lipid profile, and hypercholesterolemia. Wistar rats received a high-fat (HF) diet plus 2 ml/day of either cow's milk fermented with yogurt culture (CT), camel milk fermented with yogurt culture (CAT), camel milk fermented with *Bif. longum* BB536 (CAP), mixed cow's and camel milk fermented with yogurt culture (CCAT), or cow's milk and camel milk fermented with *Bif. longum* (CCAP). All fermented milk products significantly reduced HDL, albumin, and total protein. The percentage change in body weight gain was between −40% (CAP) and −24% (CT) and in serum triglycerides between −54% (CCAP) and −37% (CT); for the other parameters, changes caused by CCAP/CT were −40%/−22% (total cholesterol), +29%/+8% (HDL), −73%/−54% (LDL), −54%/−37% (VLDL), −52%/−14% (AST), −53%/−31% (ALT), +43%/+25% (albumin), +37%/+25% (total protein), −48%/−27% (urea), and −34%/−16% (creatinine). Camel or cow's milk fermented with yogurt culture or *Bif. longum* significantly improved negative effects of the HF diet on body weight, blood lipid profile, serum proteins, liver and kidney markers, and severity of the metabolic syndrome. Milk and fermentation culture acted synergistically with camel milk and *Bif. longum* generally showed stronger positive effects./

## 1. Introduction

Camel (*Camelus dromedarius* L.) milk only plays a minor role worldwide, but it is the most consumed milk in the Arab Gulf countries as a whole. In terms of its composition and its low coagulability, it differs significantly from cow's milk. However, the composition of camel milk varies considerably, depending on the geographical origin of the milk examined, racial, seasonal, or physiological variations, feeding, health status or physiological stage of the animals, and analytical weaknesses [1, 2]. Fat content is between 2.2 and 4.2%, of which 51–63% are saturated and 31–44% are mono- and polyunsaturated fatty acids. On average, the fat content is only slightly below that of cow's milk, whereas the cholesterol concentration of camel milk fat (34.5 mg/100 g) is higher as compared to bovine milk fat (25.63 mg/100 g) [3]. Camel milk has up to 3.5 times higher vitamin C content (51 mg/L) than cow's milk (15 mg/L), possibly to compensate for the lower vitamin E concentrations compared to cow's milk. Camel milk contains no *β*-lactoglobulin [4] and a different *β*-casein [5] and can therefore be consumed by people with cow's milk allergies. Camel milk also contains antioxidative proteins as well as biologically active peptides released from milk proteins either during normal digestion or during bacterial fermentation [6]. Favorable uses of camel milk in the metabolic syndrome, which is the combination of abdominal obesity, dyslipoproteinemia, and hypertension with peripheral insulin resistance as the causal defect, have often been postulated [7]. As a further consequence, increased levels of liver enzymes from damaged hepatocytes, impaired hepatic protein synthesis, and decreased albumin and total protein concentrations as well as increased levels of uric acid and creatinine due to kidney damage were observed [8–10]. The additional feeding of camel or cow's milk to healthy female albino rats had no significant effect on blood glucose, triglycerides, ALT, and AST. However, the cholesterol levels of both 100% cow's and 100% camel's milk-fed rats were significantly less than controls [11]. Administration of camel milk in addition to normal insulin treatment resulted in a significant reduction in blood sugar, HbA1c, insulin requirement, serum anti-insulin antibodies, urinary albumin, and body mass index, whereas C-peptide was increased [12, 13]. These investigations clearly show an insulinotropic effect of camel milk [14]. However, they do not provide any evidence that the insulin contained in camel milk or an insulin-like protein specific for camel milk acts as insulin substitutes [15]. The studies also do not show that, by the fact, camel milk does not form a solid coagulum in an acidic environment; it quickly passes through the stomach, serving as a protective vehicle for the contained insulin or insulin-like protein, thereby facilitating its intestinal absorption [16]. Such an insulinotropic effect has also been demonstrated in the whey protein fraction of the milk of other animals, particularly cow's milk.

Administration of so-called probiotic bacteria is considered another way to improve hypercholesterolemia and a disturbed lipid profile. Lowering excessive cholesterol levels is still not one of the best-established probiotic effects. In many studies, the design showed significant weaknesses, or cholesterol-lowering effects could only be observed for a short time (<6 months), or the effects could be demonstrated in vitro, but not in vivo or in clinical studies, respectively [17]. Above all, it must be taken into account that probiotic effects depend heavily on the study model and are furthermore strictly strain-specific so that research results in this regard cannot easily be transferred from one strain to another. Nevertheless, numerous probiotic microorganism strains have been investigated to date with cholesterol-lowering activity in vitro [18, 19], in experimental animals [20]. They are usually lactic acid bacteria, especially *Lactobacilli* (i.e., *L. plantarum, L. fermentum, L. brevis, and L*. *reuteri*), but also representatives of the *Enterococcus* genus or *Bifidobacteria*. In contrast, spontaneously fermented camel milk does not have a pronounced hypocholesterolemic effect, whereas Gariss containing *Bif*. *lactis* has been described to lower cholesterol concentrations in blood [21] and liver of rats [22]. Possible mechanisms of the hypocholesterolemic effect of probiotics include (a) removing of intestinal cholesterol by bacterial cells [23], (b) increase in fecal excretion of bile salts after their deconjugation by bile salt hydrolase of bacterial cells [24], and (c) inhibition of intestinal cholesterol transport and absorption by downregulation of intestinal cholesterol transport proteins (e.g., NPC1L1) [25] or due to interactions between bioactive peptides derived from camel milk proteins and cholesterol [26]. The aim of the present study is to investigate synergistic health effects of camel milk (compared to or in combination with cow's milk) and a probiotic fermentation culture (here *Bif. longum* BB536, compared to traditional yogurt) in nondiabetic rats with diet-induced obesity, impaired lipid profile, and hypercholesterolemia.

## 2. Materials and Methods

### 2.1. Materials and Kits

The reagents for the determination of serum albumin, total protein, triglycerides, total cholesterol, LDL, HDL, ALT, AST, urea, and creatinine were purchased from ELIPSE, United Diagnostic Industry (UDI), Dammam, KSA. All other chemicals were obtained from Sigma-Aldrich Chime SARL, Lyon, France.

### 2.2. Animals and Diets

Forty-nine male Wistar rats (150–180 g) were purchased from the Faculty of Pharmacy, King Saud University, KSA, and divided randomly into six groups (7 rats/group). Animals were housed in the control housing unit and were kept under standard temperature degrees and humidity (at 25°C, 55% humidity, and in a 12 : 12 h light: dark cycle) and allowed to acclimatize to the laboratory environment for one week. The composition of the basal rat diet (BD), which was obtained from the Faculty of Pharmacy, King Saud University, is shown in Table 1 in accordance with the AIN-93 guidelines [27]. The high-fat diet was prepared by adding 31.75 g animal fat, 1% cholesterol, and 0.25% bile acids (w/w) to every 67.5 g of basal diet and had the composition shown in Table 1. The guidelines of the Saudi National Committee of Bio & Med Ethics for laboratory animal care were assumed for managing the laboratory animals, and ethical admiration was concerned from the Research Ethics Committee at Qassim University, KSA [28].

#### 2.2.1. Starter Cultures and Fermented Milk Manufacture

Fresh cow and camel milk were obtained from the farm of the College of Agriculture & Veterinary Medicine, Qassim University, KSA. Fresh cow's and camel milk were mixed and homogenized for 2 min in a ratio of 50%: 50% using a high-speed mixer (2400 rpm/min), heated in a water-bath for 10 min at 85 ± 1°C, and cooled to 42 ± 1°C.

**Figure 1:**
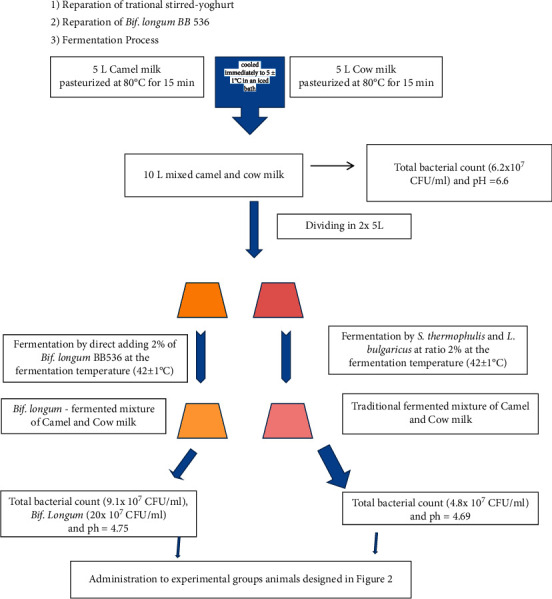
The basic process for making *Bifidobacterium longum* BB536-fermented mixture of camel and cow's milk.

*Bif. longum* BB536 and a standard yogurt culture containing a 1 : 1 mixture of *Streptococcus thermophilus* and *Lactobacillus delbrückii* ssp. *bulgaricus* were obtained from Christian Hansen (Copenhagen, Denmark). The fermented types of milk were manufactured according to [29]. Briefly, cow's (C), camel (CA), or mixed (CCA) milk were inoculated with 2% (v/v) of either traditional yogurt culture (T) or *Bif. longum* (P) and incubated at 42 ± 1°C until a pH of 4.8–4.6 was reached and the gel structure formed. The gel was immediately cooled with stirring and stored at refrigerator temperature (5 ± 1°C) until use (Figure 1).

#### 2.2.2. Chemical Analysis of Various Fermented Milk

Composition and acidity/pH of the fermented milk were investigated using customary standard methods of AOAC [30]; overall sensory quality/acceptability was tested using scoring described by [31], by 20 well-trained panelists, who had previous experience in yogurt evaluation.

### 2.3. Experimental Design

**Figure 2:**
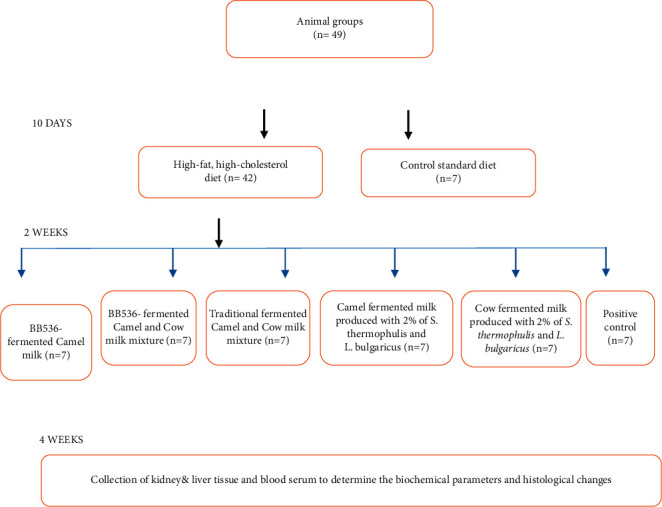
Diagram showing experimental design with animal groups.

All rats were kept under normal healthy conditions during the subsequent 2-week adaptation period and thereafter until the end of the experiment, one group of rats continued to receive the BD without further treatment, while the other animals were switched to the HF diet. During the subsequent four weeks, the second group of rats continued to receive the HF diet without further treatment until the end of the experiment, while rats in the third to seventh groups received 2 ml/day of different fermented types of milk, which were given orally by intestinal tube. At the end of the experimental period, rats were sacrificed, and then liver and kidney samples were taken surgically (3 samples/group) (Figure 2). Serum samples from all animals were obtained by centrifugation of the collected blood at 3000 rpm/10 min and stored at −20°C until analysis. To demonstrate transit tolerance and survival of *Bif. longum* BB536 in the gastrointestinal tract, stool samples were collected and aliquots were plated on MRS agar to count viable cells as previously described [32, 33].

### 2.4. Body Weight and Cumulative Weight Gain

The rats were weighed individually on the first day of the experiment. The rats were then distributed to different groups so that they were the same weight within each group. The cumulative body weight gain (cBWG) was calculated according to [34].

cBWG **=** weekly body weight gain rate − average body weight on the first day of the experiment.

### 2.5. Biochemical Analysis

Total cholesterol, HDL, and triglyceride levels were estimated in serum according to the method of [35]. LDL was calculated according to [36]. Determination of liver enzymes (ALT & AST), serum albumin, and total protein were carried out according to [37]. Kidney function was measured using serum creatinine and urea [38].

### 2.6. Histopathological Examination

Examination specimens were secured of each liver and kidney of rats and set in 10% formol saline to 24 hrs. The raised tissue parts were assembled on glass slides, deparaffinized, and dyed by hematoxylin and then eosin stain for regular testing within the light electric microscope [39].

### 2.7. Statistical Analysis

Statistical analysis was performed using the software package “Statgraphics Plus for Windows” (version 4.5, Manugistics, Rockville, MD, USA). All experiments, as well as related analysis results, were repeated three times, and the data obtained are, unless otherwise stated, presented as mean ± SE. In addition, normalized values were calculated for all parameters, in which the changes in the parameter values (vp) during the experimental phase (end value (vpe)—initial value (vpi)) of the rats that had received the HF diet plus 2 ml/day fermented milk were expressed as a percentage of the change with HF feeding only (vHFe—vHFi) according to

- **V**_**norm**_^**p**^=((*v*_*e*_^*p*^ − *v*_*i*_^*p*^) − (*v*_*e*_^HF^ − *v*_*i*_^HF^))/(*v*_*e*_^HF^ − *v*_*i*_^HF^) × 100
- **V**_**pnorm**_=(((*v*_*e*_^*HF*^ − *v*_*i*_^*HF*^)/(*v*_*e*_^*HF*^ − *v*_*i*_^*HF*^)) − 1) × 100

In order to record the overall effect of the fermented milk on the risk of a metabolic syndrome more precisely, a further parameter “metabolic syndrome risk” (MSR) was calculated separately for all diet groups in an exploratory multivariate approach. In order to determine the proportion of milk type and starter culture on the overall effect of fermented milk on the examined parameters and especially the metabolic syndrome, the parameter MSR was subjected to a 2-factor ANOVA with the factors milk type (C, CA, CCA) and starter culture (T, P).

## 3. Results

### 3.1. Suitability of the Study Model

The suitability of the animal model used for the research question was examined by comparing the BD and HF diet-fed rats (Table 2). Feeding the HF to the rats led (compared to the basal diet) to a highly significant increase in body weight gain, blood lipids, and liver enzymes between +91% and +244%. The decrease in HDL by −27% also reflects the increasing severity of the metabolic syndrome. VLDL and kidney function biomarkers are also markedly and significantly increased, while the total protein concentration in serum and serum albumin is slightly decreased.

### 3.2. Composition and Sensory Quality of Fermented Milk

The composition, pH/acidity, and overall sensory quality of the fermented milk used are listed in Table 3. The *Bif. longum* BB536 concentration in different batches of CP, CAP, and CCAP milk fluctuated but was always above 8 × 10^7^ cfu per g fermented milk product (data not shown). The data in Table 3 show that the fat and protein content as well as the overall sensory quality of the cow's milk fermented with traditional yogurt cultures was significantly higher (*p* < 0.05, 1-factor ANOVA followed by Newman-Keuls multiple comparisons) than that of the yogurt culture or *Bif. longum*-fermented camel milk (CAT, CAP). CCAT and CCAP occupy an intermediate position. For the remaining parameters, slight differences between the fermented milk are not significant and do not show any clear trends.

### 3.3. Effect of the Investigated Fermented Milk on Body Weight Gain and Metabolic Parameters in Hyperlipidemic Rats

The effect of fermented cows and camel milk on body weight, blood lipid profile, serum proteins, liver enzymes, and kidney markers in rats fed a high-fat, high-cholesterol diet is shown in Table 4. In all parameters, the investigated fermented milk products significantly reduced (increased in the case of HDL, albumin, and total protein) the values measured in rats that had not received fermented milk. The camel and cow's milk fermented with *Bif. longum* proved the most effective for all parameters; cow's milk fermented with yogurt culture was the least effective. Excessive body weight gain was most inhibited by pure camel milk fermented with *Bif. longum* BB536. The percentage change in body weight gain was between −40% (CAP) and −24% (CT), in serum triglycerides between −54% (CCAP) and −37% (CT); for the other parameters, changes caused by CCAP/CT were −40%/−22% (TC), 29/8% (HDL), −73%/−54% (LDL), −54%/−37% (VLDL), −52%/−14% (AST), −53%/−31% (ALT), 43%/25% (albumin), 37%/25% (TP), −48%/−27% (urea), and −34%/−16% (creatinine). With the exception of the HDL, albumin, and TP groups, the changes caused by CCAP or CT were significantly larger or smaller each compared with the other groups. A significant, positive overall effect on the metabolic syndrome, i.e., a reduced value of the MSR parameter, which was greatly increased by the HF diet, can also be shown in all diet groups, with the positive effect of CCAP milk being greatest (−48%) and CT milk being the least (−28%).

### 3.4. Effect of the Milk Type and Starter Culture

The share of milk type and fermentation culture in the positive overall effect of the fermented milk was determined by 2-factor ANOVA using the MSR parameter. As shown in Table 5, both the type of milk and the fermentation culture significantly influence the strength of the (positive) overall effect (global *p* < 0.05). *Bif. longum* exerts/has a significantly stronger effect than the traditional yogurt culture and both camel milk and camel milk and cow's milk influence the overall effect significantly more than cow's milk.

### 3.5. Effect of the Investigated Fermented Milk on Histological Changes in Hyperlipidemic Rats

Whereas in the rats fed the BD diet, no lesions in liver and kidney tissue were detectable, administration of the HF diet caused moderate to severe lesions in both organs (Table 6). When the hyperlipidemic rats were given fermented milk in addition to the HF diet, the symptoms were significantly weaker, or no lesions at all were observed on the liver and kidneys (Table 6). Overall, this positive effect was greatest in the rats of the CCAP group and weakest in the CT and CAT groups.

## 4. Discussion

First, the comparison of the HF with BD-fed rats (Table 2) demonstrates the suitability of the rat model used for investigating the question. Feeding the HF diet to the rats led to a highly significant increase in parameters particularly associated with the metabolic syndrome (body weight gain, blood lipids, and liver enzymes), and a decrease in HDL reflects the increasing severity of the metabolic syndrome. Therefore, rats fed the BD could be removed from further statistical analysis. Whether other factors besides milk type and fermentation culture influenced the study result (e.g., bacterial count, feed intake) cannot be said with certainty. At least the daily dose of fermented camel milk should have been sufficiently high for effectiveness because, in one of the few studies in which a dose-effect relationship was also examined [40], a daily camel milk intake in diabetic dogs was found to be higher of 100 ml as antidiabetic, which after conversion from dog to rat weight in the present study corresponds to a daily dose significantly greater than 1 ml/rat. Also, *Bif. longum* BB536 concentrations in the fermented milk always reached values that are generally believed to be sufficient for probiotic efficacy. All fermented milk investigated significantly reduced the negative (in terms of dyslipidemia and metabolic syndrome) changes caused by feeding the HF diet. In general, the mixed camel and cow's milk fermented with *Bif. longum* BB536 showed the strongest positive effects: the additional feeding of the CCAP milk restored the lipid profile of rats fed exclusively the BD to 75 to 93%. But, feeding on CT restored the lipid profile of rats fed the BD diet to a lower extend (24 to 69%). A significant, positive overall effect on the metabolic syndrome, i.e., a reduced value of the MSR parameter, which was greatly increased by the HF diet, can also be shown in all diet groups, with the positive effect of CCAP milk being the greatest (−48%) and CT milk being the least (−28%). As the results of the 2-factor ANOVA (Table 5) show, both the milk type and the starter culture make significantly different (global *p* values 0.0017 or 0.0203, respectively) independent contributions to the overall beneficial effect of fermented milk in obese hypercholesterolemic rats. Within the “milk type” factor, the beneficial effect of CAT or the CCAT is significantly stronger than that of CT, whereas the difference between CAT and CCAT milk is not significant. *Bif. longum* BB536 has a significantly stronger beneficial effect than the traditional starter culture.

The findings made here correspond to a high degree to the results of in vivo studies in which the effect of camel milk on the glycemic and lipid profile of streptozotocin- or alloxan-induced diabetic rats [41, 42] was examined. In these studies, the intake of raw, unfermented camel milk by the experimental animals significantly decreased elevated levels of serum triglycerides, total cholesterol, and LDL and VLDL and increased HDL cholesterol. In addition, oxidative stress (measured as MDA) was reduced, liver function markers (ALT, AST) were restored to 50 to 90% of control levels, hepatic protein synthesis was increased, levels of urea/uric acid and creatinine were reduced, and diabetes-associated liver and renal damage were alleviated. Also, nondiabetic rats had developed characteristics of nonalcoholic fatty liver disease due to the administration of a high-cholesterol diet (hepatic steatosis, inflammatory cellular infiltration in liver tissue, altered liver functions, increased oxidative stress, and an increase in serum triglycerides and total - LDL- and VLDL cholesterol as well as a decrease in HDL cholesterol). The additional feeding of camel milk reduced oxidative stress, hepatic steatosis, and inflammatory cell infiltration, restored altered liver functions, and normalized triglyceride and cholesterol profile [43]. In contrast, feeding camel milk to healthy, nondiabetic rats had no significant effect on blood glucose, serum triglycerides, and cholesterol compared to no treatment or administration of cow's milk [44]. In other studies, in which cow's milk-fed experimental animals were used as controls, cow's milk showed weaker positive effects than camel milk [10]. When looking for the causes of the beneficial effects of fermented cow's or camel milk on body weight and blood lipid profile, as observed in the present study on obese, hypercholesterolemic rats, one must consider milk type and fermentation culture separately, because both have independent effects, as shown in the present study.

### 4.1. Mechanisms of Camel Milk Effects on the Metabolic Syndrome

One of the main effects attributed to camel milk is its hypoglycemic, antidiabetic effect as demonstrated in numerous animal and human studies. However, obesity and the observed disorders of the lipid profile are essential characteristics of the metabolic syndrome, in which peripheral insulin resistance is widely believed to be an essential factor [45]. Characteristics of dyslipoproteinemia, which is also part of the metabolic syndrome, are hypertriglyceridemia with an increase in the VLDL concentration and hypercholesterolemia with an increase in LDL cholesterol and a decrease in HDL cholesterol [46]. This close correlation suggests a common underlying mechanism [47], which can be summarized as follows. Administration of energy, fat, and cholesterol-rich diets to the rats led to obesity, increased serum concentrations of triglycerides and free fatty acids [48], and resulted in increased insulin resistance in the animals [46], combined with compensatory hyperinsulinemia. The latter, mediated by the transcription factor SREBP1-c, increases fatty acid and triglyceride synthesis and thus promotes the formation and release of triglyceride-rich VLDL particles in the liver, as does the increased supply of triglycerides and FFA from dietary fats. Metabolism of VLDL in peripheral tissues is dependent on endothelial lipoprotein lipase, which has been shown to have reduced activity by insulin in metabolic syndrome [49]. Thus, in the presence of insulin resistance, plasma VLDL concentration is increased by increased VLDL synthesis in the liver and decreased peripheral metabolization. This leads to increased degradation in the circulation to intermediate-density lipoproteins and ultimately to LDL particles [49], and it has been shown that any increase in triglyceride-rich VLDL particles results in increased formation of cholesterol-rich LDL particles [50]. The lower HDL concentration can also be explained by insulin resistance [51]. HDL results from the degradation of VLDL by lipoprotein lipase [52], which may explain a decreased HDL concentration in peripheral blood under the conditions of reduced lipoprotein lipase activity in insulin resistance. Most importantly, the beneficial effects of camel and cow's milk observed in the study can be easily explained by a reduction in insulin resistance due to the insulinotropic effect of milk and especially camel milk [53].

### 4.2. Mechanisms of Positive Effects on Liver and Renal Damage

In addition to increased hyperglycemia, which is believed to be the main cause of liver and kidney damage associated with experimental diabetes in rats [10], liver atherosclerosis, lipid peroxidation, and abnormalities in lipid metabolism in rats fed on an HF diet are other major causes of liver and renal damage [54, 55]. Hypercholesterolemia may lead to increased lipid peroxidation, particularly of cell membranes, to tissue damage and to the production of several degeneration products, which may reduce cell replication and cell life. The resulting increased damage to hepatocytes and loss of liver functionality leads to increased release and concentration of ALT and AST in the blood and decreased protein synthesis in the liver, while kidney damage is the cause of increased serum concentrations of urea and creatinine [11, 54]. The present study confirmed the negative effect of the HF diet in rats, so one can assume that the partial reversal of these effects due to the administration of milk is partly due to their antioxidant content and the stronger effect of camels in comparison to cow's milk is a result of their higher antioxidant content [56].

### 4.3. Effects of Fermentation Cultures/Probiotics

In the past few decades, numerous mechanisms have been proposed and investigated as the cause of a hypolipidemic and particularly hypocholesterolemic effect of certain probiotic *Lactobacilli* and *Bifidobacteria* but also other genera including *Enterococcus* and *Bacteroides* [18, 19]. The fact that diverse and sometimes contradicting results were often obtained in different studies may be related to the pronounced strain specificity of probiotic effects (e.g., its bile salt hydrolase BSH, activity) to the type of experiments (in vitro investigations or animal and human studies in vivo), other experimental conditions (pH value, bile acids added, resting or growing bacteria, etc.), and the viability of the probiotics ingested and their ability to colonize the small intestine. Although there are still many unanswered questions, it seems that having a bile salt hydrolase and the resulting ability to hydrolyze glyco- and tauroconjugated bile salts to glycine/taurine and free (deconjugated) bile salts is the key factor responsible for lowering the cholesterol level by probiotic bacteria [57]. Deconjugated bile salts are less efficiently reabsorbed than their conjugated counterparts and are therefore excreted to a greater extent in the feces. They are also adsorbed to a greater extent to bacterial cells and dietary fiber in the intestine, which may also contribute to an increased fecal excretion [58]. Both have the effect that more bile salts are withdrawn from the enterohepatic circulation, which is replaced in the liver by bile salts newly synthesized from cholesterol, which in turn leads to lowered serum cholesterol levels. Moreover, in the presence of Ca^2+^ or at pH values below 5.5 to 6, deconjugated bile acids precipitate more easily than conjugated ones alone or as a coprecipitate with cholesterol [18]. In men, this effect is likely to be of limited importance, because the human small intestine has a neutral to alkaline pH (>6). In rats, however, the intestinal pH is significantly lower, in fed animals around 5.0 in duodenum and jejunum, just below 6 in ileum and caecum, and between 5.5 and 6 in the colon [59]. At pH values above 6, a cholesterol reduction from the culture media by some probiotics should not be based on coprecipitation, but rather on assimilation, i.e., the uptake of cholesterol in the cell membrane of bacteria [60]. Finally, the reduced availability of bile salts due to microbial deconjugation leads to impaired cholesterol absorption from the intestinal lumen, since cholesterol must be soluble to be absorbed and its solubilization occurs in the form of mixed micelles of cholesterol, phospholipids, and available bile salts [32].

*Streptococcus thermophilus* has BSH activity that, depending on the subspecies, is weaker or greater than that of effective probiotics (*L. plantarum* XJ-14, *L. rhamnosus* GG) or comparable to this [61]. The weaker hypocholesterolemic effect of conventional yogurt culture, as in the present study or in a study on hypercholesterolemic subjects [62], should therefore come from the acid sensitivity of *S. thermophilus* [63], and/or the lower cholesterol assimilation of *S*. *thermophilus* or a conventional yogurt culture [19]. Other mechanisms that could contribute to the demonstrated potential of probiotic bacteria to lower cholesterol levels include conversion of cholesterol to coprostanol by small intestinal and colonic bacteria, leading to enhanced fecal excretion and a reduction in the total physiological concentration of cholesterol [23], and fermentation of indigestible food carbohydrates to short-chain fatty acids (especially propionate), which can lower blood lipids through blocking the synthesis of hepatic cholesterol and/or through redirecting plasma cholesterol toward the liver [64]. The decrease in serum triglycerides caused by certain probiotics in parallel with the decrease in VLDL cholesterol and LDL cholesterol and the increase in HDL-C has been shown in a number of studies, but the underlying mechanisms are much less investigated than with cholesterol [65, 66]. The relationship between bile salt and triglyceride/lipoprotein metabolism is likely to play an important, albeit still largely not understood, role. The suppression of the VLDL secretion of human hepatocytes by bile acids could be shown, and a physiological function of the enterohepatic circulation of bile acids in the regulation of postprandial lipid concentrations was postulated [67]. Other mechanisms that could lead to a bacteria-mediated decrease in the plasma triglyceride concentration include alterations in the regulation of lipogenic enzyme activities [41] or upregulation of PPAR*α*, bile acid receptor (FXR), and ApoA-V [68].

In summary, it can be concluded from the facts that *Bif. longum* has a relevant BSH activity [19] and the pH value in the small intestine of rats is below 5.5 and can possibly drop even further due to the fermentation activity of *Bif. longum* that, in the present study, the hypocholesterolemic effect of BB536 and the weaker hypocholesterolemic effect of the yogurt culture are primarily based on bile salt deconjugation, impaired intestinal absorption, and increased fecal excretion of bile acids and the increased cholesterol requirement of the liver for the synthesis of new bile acids. This view is supported by studies in which feeding a *Bif. longum* BB536-containing yogurt to rats in addition to a high-cholesterol diet not only lowered plasma TC, LDL-C, and VLDL-C but also significantly increased fecal excretion of bile acids [55, 69]. In a further study [57], the authors showed that, in a taurocholic acid-containing culture medium, growing *Lactobacillus* and *Bifidobacterium* species removed cholesterol both by bacterial assimilation/uptake and by precipitation, while in the presence of Oxgall this was only for *Lactobacilli,* while—as assumed in the present study—only precipitation occurred for Bifidobacteria.

## 5. Conclusions

*Bif. longum* BB536 exerts/has a significantly stronger effect than the traditional yogurt culture and both camel milk and camel milk plus cow's milk influence the overall effect significantly more than cow's milk. Camel milk fermented with *Bif. longum* BB536 considerably reduces the risk of dyslipidemia associated with the metabolic syndrome in obese, hypercholesterolemic rats, by reducing body weight gain and the serum levels of triglycerides, VLDL, and LDL and increasing serum HDL. Kidney function biomarkers (urea, creatinine) are also markedly and significantly increased, while the total protein concentration in serum and serum albumin is slightly decreased. These metabolic improvements go hand in hand with the reduction of tissue damage (liver, kidney).

## Figures and Tables

**Table 1:** Effect of the high-fat diet compared to the basal diet on body weight gain and metabolic parameters in male albino rats.

Ingredient	Diet	
	Basal diet	High-fat diet (%)
Casein	20%	13.4
Fat	5%^1^	35.1^2^
Sucrose	23%	15.4
Corn starch	15%	10.1
Cellulose	5%	3.4
Soybean	24%	16.1
Cholesterol	—	1.0
Bile acids	—	0.3
Mineral mix	6%	4.0
Vitamin mix	1%	0.7
DL-methionine	0.7%	0.5
Choline bitartrate	0.3%	0.2

^1^Corn oil. ^2^Corn oil + animal fat, 1 : 9.5.

**Table 2:** Effect of the high-fat diet compared to the basal diet on body weight gain and metabolic parameters in male albino rats.

Parameters	Basal diet	High-fat diet	Change (%)	*p*
Body weight gain (%)	13.2 ± 0.2	34.0 ± 1.6	+157	<0.01
TG (mg/dl)	45.6 ± 1.6	156.1 ± 4.1	+242	<0.01
TC (mg/dl)	74.6 ± 3.7	142.4 ± 6.2	+91	<0.01
HDL (mg/dl)	37.7 ± 0.9	27.5 ± 0.4	−27	<0.01
LDL (mg/dl)	28.2 ± 1.5	133.7 ± 1.8	+374	<0.01
VLDL (mg/dl)	9.1 ± 0.1	31.3 ± 0.6	+244	<0.01
AST (U/L)	43.7 ± 1.9	115.3 ± 1.6	+164	<0.01
ALT (U/L)	24.4 ± 0.6	60.0 ± 1.0	+146	<0.01
Albumin (g/dl)	3.8 ± 0.2	3.0 ± 0.1	−21	n.s.
Total protein (g/dl)	7.3 ± 0.1	5.5 ± 0.1	−25	<0.01
Urea (mg/dl)	14.5 ± 0.1	37.9 ± 0.1	+161	<0.01
Creatinine (mg/dl)	0.9 ± 0.1	1.6 ± 0.1	+77	<0.01

**Table 3:** Composition and sensory quality of different fermented milk products.

Parameters	Fermented milk types				
	CT	CAT	CAP	CCAT	CCAP
Fat (%)	4.80 ± 0.33^a^	3.3 ± 0.16^b^	3.50 ± 0.20^b^	4.35 ± 0.12^a^	4.50 ± 0.33^a^
Protein (%)	4.83 ± 0.21^a^	4.39 ± 0.08^b^	4.40 ± 0.10^b^	4.47 ± 0.14^ab^	4.49 ± 0.24^ab^
Ash (%)	0.78 ± 0.07^a^	0.86 ± 0.10^a^	0.84 ± 0.10^a^	0.80 ± 0.08^a^	0.82 ± 0.09^a^
Lactose (%)	3.93 ± 0.17^a^	3.97 ± 0.22^a^	3.89 ± 0.32^a^	3.76 ± 0.16^a^	3.73 ± 0.14^a^
T.S (%)	14.34 ± 0.31^a^	12.52 ± 0.27^c^	12.63 ± 0.21^c^	13.38 ± 0.19^b^	13.54 ± 0.23^b^
pH	4.83 ± 0.10^a^	4.73 ± 0.13^a^	4.73 ± 0.12^a^	4.75 ± 0.12^a^	4.69 ± 0.09^a^
Acidity (%)	0.78 ± 0.11^a^	0.71 ± 0.09^a^	0.74 ± 0.11^a^	0.75 ± 0.11^a^	0.73 ± 0.11^a^
Overall acceptability (out of 10 points)	9.31 ± 0.13^a^	8.24 ± 0.17^cd^	8.15 ± 0.12^d^	8.9 ± 0.21^b^	8.6 ± 0.23^bc^

Different superscript letters (a to c) within the same raw showed significant differences among the groups (*p* ≤ 0.05). CT: fermented cow's milk with a traditional starter culture; CAT: fermented camel milk with a traditional starter culture; CAP: fermented camel milk with *Bif. longum* BB536; CCAT: fermented camel plus cow's milk with a traditional starter culture; CCAP: fermented camel plus cow's milk with *Bif. longum* BB536.

**Table 4:** Effect of various fermented types of milk on body weight, blood lipid profile, serum proteins, liver enzymes, and kidney markers in overweight, hypercholesterolemic-made rats on a high-fat, high-cholesterol diet.

Rat group	HF diet only	HF diet plus					*p*
Parameter		CCAP	CAP	CCAT	CAT	CT	
A. Body weight							
BW gain % (13)	34.0 ± 1.62^a^	22.35 ± 0.9^c^	20.34 ± 0.3^d^	24.78 ± 0.7^b^	22.3 ± 0.5^c^	26.01 ± 0.8^b^	≤0.00

B. Blood lipid profile							
TG mg/dl (46)	156.1 ± 4.1^a^	71.5 ± 2.8^d^	80.9 ± 1.7^c^	80.08 ± 2.8^c^	83.3 ± 2.5^c^	98.0 ± 3.3^b^	≤0.00
TC mg/dl (75)	142.4 ± 6.0^a^	85.5 ± 2.2^e^	93.1 ± 1.6^cd^	91.27 ± 5.1^c^	98.9 ± 2.26^c^	110.3 ± 3.6^b^	≤0.00
HDL mg/dl (38)	27.5 ± 0.41^d^	35.5 ± 2.2^ab^	33.1 ± 1.9^bc^	32.9 ± 0.55^bc^	30.4 ± 2.9^cd^	29.8 ± 1.35^cd^	≤0.006
LDL mg/dl (28)	133.7 ± 1.8^a^	35.7 ± 2.7^e^	43.8 ± 1.8^d^	42.6 ± 2.3^c^	41.9 ± 1.7^c^	60.9 ± 1.7^b^	≤0.00
VLDL mg/dl (9)	31.27 ± 0.6^a^	14.3 ± 2.7^d^	16.2 ± 0.3^c^	15.32 ± 0.6^c^	16.5 ± 0.4^c^	19.6 ± 0.6^b^	≤0.00

C. Liver function							
AST (U/L) (44)	115.3 ± 1.6^a^	55 ± 0.73^f^	63.3 ± 0.6^e^	80.3 ± 0.63^c^	65.7 ± 1.0^d^	99.3 ± 0.65^b^	≤0.00
ALT (U/L) (24)	60.0 ± 1.0^a^	28.3 ± 0.63^f^	33.9 ± 0.9^d^	32.1 ± 0.7^c^	35.6 ± 0.7^c^	41.7 ± 0.42^b^	≤0.00
Albumin (g/dl) (4)	2.95 ± 0.03^e^	4.22 ± 0.14^a^	4.1 ± 0.09^ab^	3.93 ± 0.13^bc^	3.91 ± 0.1^bcd^	3.7 ± 0.02^d^	≤0.00
TP (g/dl) (7)	5.48 ± 0.12^f^	7.56 ± 0.08^a^	7.5 ± 0.11^ab^	7.10 ± 0.09^de^	7.31 ± 0.1^bc^	6.9 ± 0.06^e^	≤0.00

D. renal function							
Urea (mg/dl) (15)	37.89 ± 0.1^a^	19.75 ± 0.26^f^	22.2 ± 0.12^e^	23 ± 0.16^d^	25.1 ± 0.36^c^	27.57 ± 0.3^b^	≤0.00
Creatinine (mg/dl)	1.57 ± 0.11^a^	1.05 ± 0.05c^d^	1.07 ± 0.7^cd^	1.11 ± 0.09^c^	1.16 ± 0.12^bc^	1.34 ± 0.11^b^	≤0.001

E. Metabolic syndrome^1^							
Δ MSR (%)	0.0005 ± 1.37^a^	−47.5 ± 1.37^b^	−42.46 ± 1.37^c^	−39.11 ± 1.37^c^	−38.66 ± 1.37^c^	−27.69 ± 1.37^d^	≤0.00

^**1**^BWG + TG + TC-HDL + VLDL + ALT + AST after normalization of the data.

**Table 5:** Effect of the milk type and starter culture on the improvement of parameters associated with the metabolic syndrome by fermented milk in 105 hypercholesterolemic rats.

Parameter^1^	*n* ^2^	LS means^3^	Stand. error	Global *P*	Significantly different from
*Milk type*					
C	21	−30.74	2.90	0.0017	CA, CCA
CA	42	−40.56	1.83		C
CCA	42	−43.32	1.83		C

*Fermentation culture*					
P	42	−41.26	2.11	0.0203	T
T	63	−35.16	1.50		P

^1^C: cow's milk; CA: camel milk; CCA: cow's plus camel milk; B: *Bif. longum* BB536; T: traditional yogurt culture; ^2^*n*: number of measured cases; ^3^LS means: least squares means (computed means based on a linear model (here ANOVA)).

**Table 6:** Effect of different fermented milk products on histological changes of the liver and kidney in hypercholesterolemic rats.

Organs	Lesions	BD	HF	CT	CAT	CAP	CCAT	CCAP
Liver	Steatosis	−	+++	−	−	−	−	−
	Congestion	−	++	+	+	+	+	+
	Portal inflammation	−	++	+	+	+	−	−
	Newly formed canaliculi	−	++	−	−	−	−	−
	Kupffer cells hyperplasia	−	++	+	+	+	+	−
	Adipocytes	−	−	−	−	−	−	−

Kidney	Shrunken glomeruli	−	+++	−	−	−	−	−
	Degenerated renal tubules	−	+++	+	+	+	+	−
	Congestion	−	++	+	+	+	+	−
	Casts	−	+++	−	−	−	−	−
	Lymphocytic infiltrations	−	+++	−	−	−	−	+
	Hypercellularity of glomeruli	−	−	+	+	−	−	+

BD: basal diet; HF: high-fat, high-cholesterol diet {− = no or within the normal limit (0%), + = mild alterations (10–25%), ++ = moderate alterations (30–45%), +++ = severe alterations (up to 50%)}.

## Data Availability

The data used to support the findings of this study are included within the article.

## Additional Points

Camel milk fermented with *Bifidobacterium longum* BB536 reduces the risk of metabolic syndrome in obese, hypercholesterolemic rats by reducing body weight gain and the serum levels of triglycerides, VLDL, and LDL and increasing serum HDL. These metabolic improvements go hand in hand with the reduction of tissue damage (liver and kidney).
